# Novel small molecule inhibition of IKK/NF‐κB activation reduces markers of senescence and improves healthspan in mouse models of aging

**DOI:** 10.1111/acel.13486

**Published:** 2021-11-03

**Authors:** Lei Zhang, Jing Zhao, Xiaodong Mu, Sara J. McGowan, Luise Angelini, Ryan D. O'Kelly, Matthew J. Yousefzadeh, Ayumi Sakamoto, Zaira Aversa, Nathan K. LeBrasseur, Yousin Suh, Johnny Huard, Theodore M. Kamenecka, Laura J. Niedernhofer, Paul D. Robbins

**Affiliations:** ^1^ Department of Biochemistry, Molecular Biology and Biophysics Institute on the Biology of Aging and Metabolism University of Minnesota Minneapolis Minnesota USA; ^2^ Department of Molecular Medicine Scripps Research Jupiter Florida USA; ^3^ Center for Regenerative Sports Medicine Steadman Philippon Research Institute Vail Colorado USA; ^4^ Shandong First Medical University (Shandong Academy of Medical Sciences) Jinan China; ^5^ Department of Physical Medicine and Rehabilitation and Robert and Arlene Kogod Center on Aging Rochester Minnesota USA; ^6^ Department of Genetics and Development Columbia University New York New York USA

**Keywords:** aging, NEMO, NF‐κB, senescence, SR12343

## Abstract

Constitutive NF‐κB activation is associated with cellular senescence and stem cell dysfunction and rare variants in NF‐κB family members are enriched in centenarians. We recently identified a novel small molecule (SR12343) that inhibits IKK/NF‐κB activation by disrupting the association between IKKβ and NEMO. Here we investigated the therapeutic effects of SR12343 on senescence and aging in three different mouse models. SR12343 reduced senescence‐associated beta‐galactosidase (SA‐β‐gal) activity in oxidative stress‐induced senescent mouse embryonic fibroblasts as well as in etoposide‐induced senescent human IMR90 cells. Chronic administration of SR12343 to the *Ercc1* ^−/^ *^∆^* and *Zmpste24* ^−/−^ mouse models of accelerated aging reduced markers of cellular senescence and SASP and improved multiple parameters of aging. SR12343 also reduced markers of senescence and increased muscle fiber size in 2‐year‐old WT mice. Taken together, these results demonstrate that IKK/NF‐κB signaling pathway represents a promising target for reducing markers of cellular senescence, extending healthspan and treating age‐related diseases.

## Graphical abstract

Constitutive IKK/NF‐κB activation is associated with cellular senescence and aging. Here we demonstrate that pharmacological inhibition of IKK/NF‐κB activation by the small molecule SR12343 reduced senescence and SAPS factors in cell culture, improved healthspan in mice with accelerated aging, and reduced markers of senescence and pathology in multiple tissues of both accelerated and naturally aged mice. Therefore, IKK/NF‐κB pathway is a promising target for aging interventions to treat age‐related diseases.

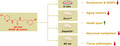

**Abbreviations**
NEMO: NF‐κB essential modulator
SASP: senescence‐associated secretory phenotype
NBD: NEMO Binding Domain
SA‐β‐gal: senescence‐associated beta‐galactosidase
NMR: nuclear magnetic resonance
OGTT: oral glucose tolerance test
AUC: area under the curve
MPC: Muscle progenitor cell
MSC: mesenchymal stem cell
f‐MyHC: fast‐type myosin heavy chain
MEF: mouse embryonic fibroblast

## 1 INTRODUCTION

NF‐κB is an inducible transcription factor comprised of five different family members, RelA/p65, RelB, c‐Rel, p50/p105, and p52/p100, capable of regulating diverse biological processes, including inflammation, immunity, stress responses, cell proliferation, differentiation, and survival (Zhang et al., 2017). A wide range of external, internal, and environmental inducers can activate the NF‐κB signaling including growth factors, viral or pathogenic assaults, tissue injury, genotoxic, oxidative and inflammatory stresses. Generally, these signals initiate the upstream NF‐κB signaling cascades via three different pathways: the canonical, non‐canonical, and atypical (Zhang et al., 2019). Subsequent signaling cascades in most cases converge on the IKK complex formed by two catalytic subunits, IKKα and IKKβ, and a regulatory subunit IKKγ (NEMO). Activated IKK complex phosphorylates IκB proteins, leading to its subsequent degradation. As a result, the NF‐κB dimer sequestered in the cytoplasm is liberated and translocated to the nucleus to activate specific transcriptional machinery. Normal activation of NF‐κB is required to maintain many physiological functions, whereas its abnormal or chronic activation have been linked to many inflammatory and age‐related diseases (Amiri & Richmond, 2005; Baker et al., 2011).

NF‐κB also plays key roles in cellular senescence and the aging process (Salminen & Kaarniranta, 2009; Tilstra et al., 2011). Cellular senescence is an irreversible growth arrest in response to different stresses and one defining feature of senescent cells is the secretion of a mix of factors termed the senescence‐associated secretory phenotype (SASP), comprised of pro‐inflammatory mediators, growth factors, metalloproteinases and other components (Gorgoulis et al., 2019). Diverse stress conditions contribute to the progression of senescence and aging, such as oxidative, metabolic, and genotoxic stresses. Chronic inflammation, a hallmark of aging (Lopez‐Otin et al., 2013), also induces cellular senescence and promotes tissue aging. Bioinformatics studies demonstrated that NF‐κB is the transcription factor most associated with mammalian aging (Adler et al., 2007). Furthermore, constitutive NF‐κB activation drives senescence and mammalian aging, conferring expression of SASP factors including pro‐inflammatory cytokines and chemokines (e.g., p16^INK4a^, p21^CIP1^, IL‐6, IL‐1α and TNFα) (Bernard et al., 2004; Osorio et al., 2016; Salminen et al., 2012; Seitz et al., 2000; Zhi et al., 2011). The elevated production of SASPs can further enhance cellular senescence in cell autonomous and/or cell non‐autonomous pathways, contributing to many pathological disorders associated with aging (Acosta et al., 2013; Chien et al., 2011; Coppe et al., 2010; Nelson et al., 2012). For example, overexpression of c‐Rel in normal young keratinocytes induced senescent cellular phenotypes including decreased proliferation, apoptosis resistance, enlargement and polynucleation (Bernard et al., 2004). Moreover, acute genetic blockade of NF‐κB signaling in epidermis of old mice reduced the expression of age‐associated genes and reverted many features of aging to that observed in young mice (Adler et al., 2007). We and others also demonstrated that inhibition of NF‐κB by genetic depletion of one allele of the p65 subunit of NF‐κB delayed the onset of aging‐related symptoms and extended healthspan in *Sirt6* ^−/−^, *Ercc1* ^−/^ *^Δ^* and *Zmpste24* ^−/−^ progeroid mice (Kawahara et al., 2009; Osorio et al., 2012; Tilstra et al., 2012). Finally, we recently have identified rare variants in three different NF‐κB family members, the transcription factors p65 (*RELA*) and p50 (*NFKB1*) and the repressor IκBα (*NFKBIA*), enriched in centenarians and associated with longevity (Ryu et al., 2021). These studies convincingly established a causal role of NF‐κB activation in natural and accelerated aging.

Given its key role in senescence and aging, the NF‐κB signaling pathway presents a therapeutic target for extending healthspan (Salminen & Kaarniranta, 2009; Tilstra et al., 2011). Previously, we developed the small molecule SR12343 capable of inhibiting NF‐κB activation by disrupting the association between IKKβ and NEMO (Zhao et al., 2018). SR12343 was developed to act as a mimetic of the NEMO Binding Domain (NBD) peptide shown previously to improve pathology in many pre‐clinical models as well as to reduce senescence and improve healthspan in a mouse model of accelerated aging (Tilstra et al., 2012). Treatment with SR12343 demonstrated positive effects in murine models of acute inflammation and in the *mdx* mouse model of Duchenne muscular dystrophy (Zhao et al., 2018). Here, we examined the therapeutic potential of SR12343 in reducing cellular senescence and extending healthspan using both cell‐based models and mouse models of accelerated and naturally aging.

## 2 RESULTS

### 2.1 SR12343 reduces markers of cellular senescence

**FIGURE 1:**
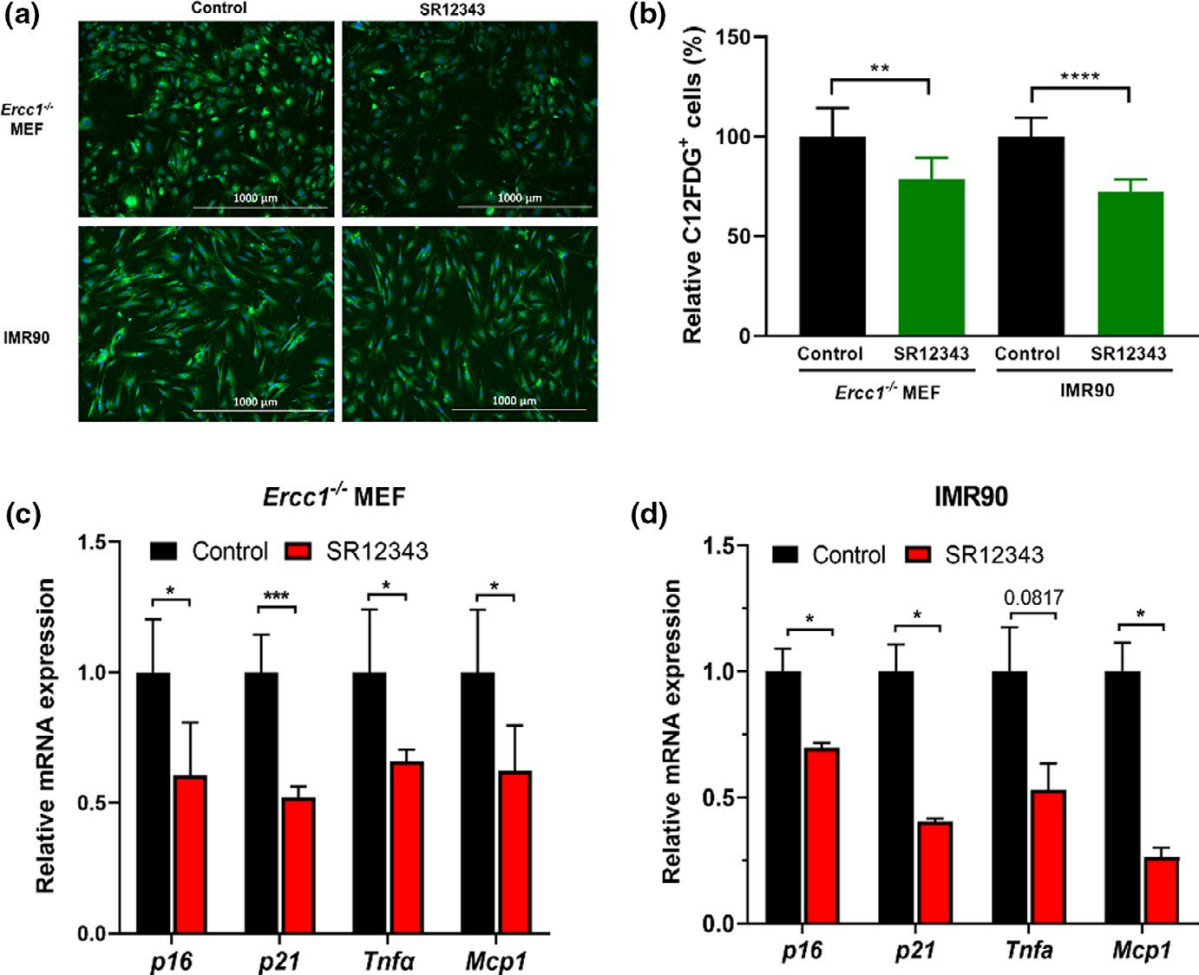
SR12343 reduces cellular senescence in both mouse and human cells. (a) Representative images of SA‐β‐gal senescence assay by C_12_FDG staining in senescent *Ercc1* ^−/−^ MEFs and IMR90 cells with or without treatment of SR12343 (50 µM), where blue fluorescence indicates DNA staining with Hoechst 33324 and green fluorescence indicates SA‐β‐gal staining with C_12_FDG. Senescence was induced by oxidative stress (20% O_2_, MEFs) and genotoxic stress (etoposide, IMR90). (b) Quantification of C_12_FDG senescence assay. Error bars indicate SEM for *n* = 3. RT‐qPCR analysis of expression of senescence biomarkers in senescent (c) *Ercc1* ^−/−^ MEFs and (d) IMR90 cells with or without treatment of SR12343 (50 µM). Error bars indicate SEM for *n* = 4

To determine if SR12343 reduces markers of cellular senescence, similar to other IKK/NF‐κB inhibitors, senescent murine *Ercc1*‐deficient MEFs and human IMR90s were generated as described (Yousefzadeh et al., 2018) and treated with SR12343. SR12343 significantly reduced the number of C_12_FDG positive cells (Figure 1a,b) without cytotoxicity 48 h post‐treatment (Figure S1a). As determined by RT‐qPCR, SR12343 treatment significantly reduced the expression of critical senescence‐associated marker genes in both senescent MEFs and IMR90 cells, including *p16^Ink4a^*, *p21^Cip1^*, *Tnfα* and *Mcp1* (Figure 1c,d). Moreover, SR12343 could inhibit cell proliferation as its treatment reduced the percentage of EdU positive cells compared with the control (Figure S1b,c). Interestingly, SR12343 inhibited the expression of senescence related genes including *p16^Ink4a^*, *p21^Cip1^*, and *p53* whereas increased the gene expression of cell cycle regulators such as *p15* or *p27* (Figure S1d), indicating that SR12343 is able to reduce cellular senescence and SASP factors while mediating the cell cycle arrest. Collectively, these observations demonstrate that SR12343 functions as a senomorphic to reduce expression of markers of cellular senescence and SASP in both mouse and human senescent cells.

### 2.2 SR12343 extends healthspan in progeroid model of *Ercc1* ^−/Δ^ mice

**FIGURE 2:**
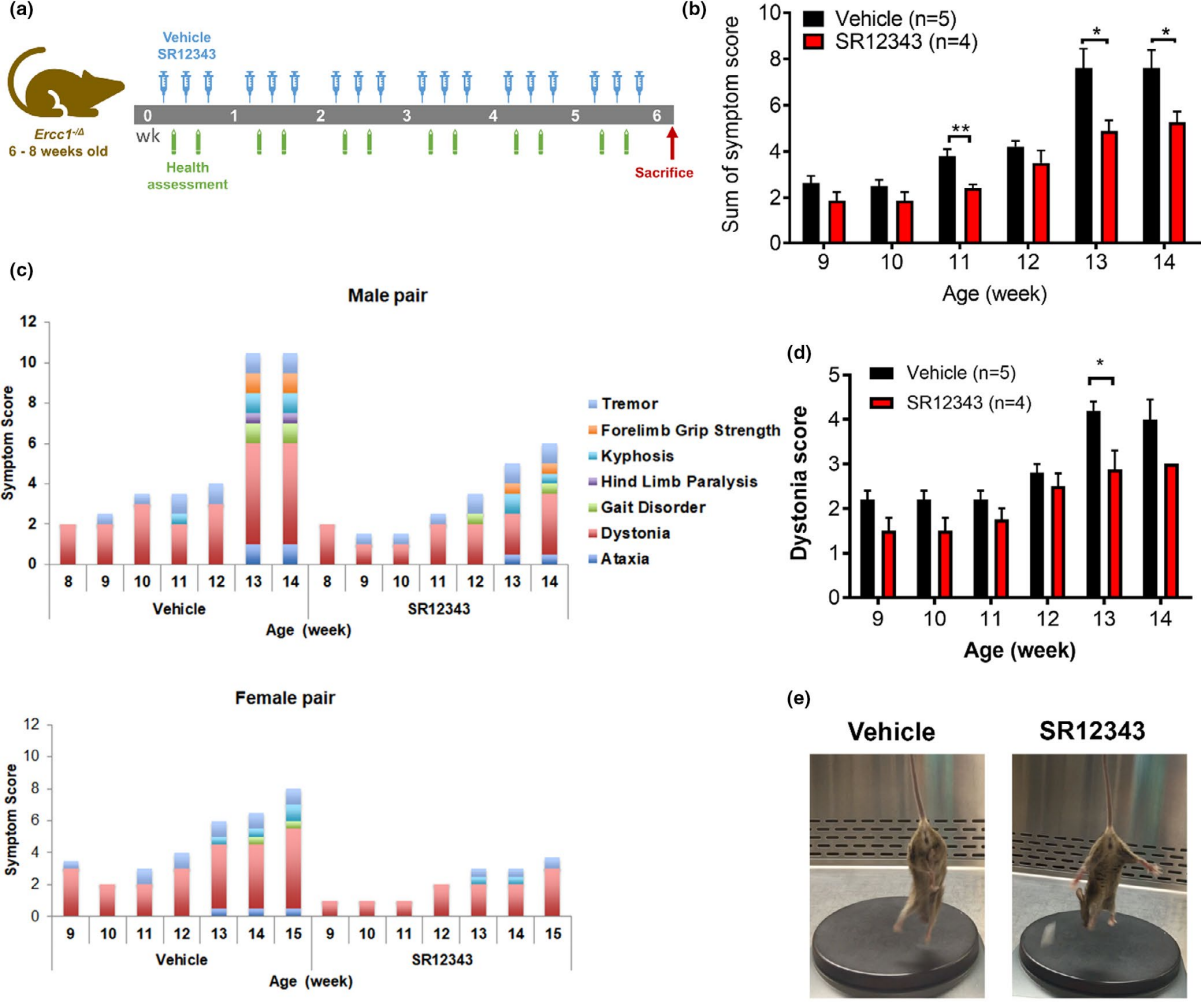
Chronic treatment with IKK/NF‐κB inhibitor SR12343 extends healthspan in *Ercc1* ^−/^ *^Δ^* mice. (a) *Ercc1* ^−/^ *^Δ^* mice were treated intraperitoneally with 30 mg/kg of SR12343 or vehicle three times per week, starting from 6 to 8 weeks of age. Mice were sacrificed at 15 weeks of age and tissues collected for analysis. (b) The sum of the symptoms scores of *Ercc1* ^−/^ *^Δ^* mice reflects the general health condition, neurodegeneration and muscle wasting. Error bars indicate SE. (c) Pair‐based plotting of aging symptoms. Higher stacking score indicates early onset and/or rapid progression of aging symptoms. (d) Dystonia was measured twice per week and scored based on the severity and the time required for the pathological contraction. Error bars indicate SE. (e) Representative images showing attenuated dystonia after SR12343 treatment

To examine the ability of SR12343 to suppress markers of senescence and aging *in vivo*, we initially utilized the *Ercc1* ^−/Δ^ mouse, a model of accelerated aging that mimics human XFE progeria. This model has reduced expression of ERCC1‐XPF DNA repair endonuclease (Robinson et al., 2018) with accelerated senescence and age 6 times faster than WT mice due to their impaired capacity to repair the nuclear genome (Gurkar & Niedernhofer, 2015). Importantly, the *Ercc1* ^−/^ *^Δ^* mice have a similar pattern of senescence in different tissues as naturally aged mice (Yousefzadeh et al., 2020). The *Ercc1* ^−/^ *^Δ^* mice were dosed i.p. with vehicle or SR12343 (30 mg/kg) three times per week for 8 weeks, starting at 6–8 weeks of age. Health assessments were performed twice per week (Figure 2a) with tremor, kyphosis, dystonia, ataxia, gait disorder, hindlimb paralysis and forelimb grip strength scored separately. The composite score of all signs of aging reflects the overall health condition of *Ercc1* ^−/^ *^Δ^* mice. The *Ercc1* ^−/^ *^Δ^* mice treated with SR12343 showed reduced frailty and delayed progression of signs of aging (Figure 2b), especially between 11 and 14 weeks. A gender‐matched comparison also showed delayed onset, reduced severity, and slowed progression of aging pathologies in male and female mice (Figure 2c). Among the neurodegenerative symptoms, SR12343 treatment had the strongest effect in attenuating progression and severity of dystonia (Figure 2d,e).

### 2.3 SR12343 decreases markers of cellular senescence in the liver of *Ercc1* ^−/Δ^ progeroid mice

**FIGURE 3:**
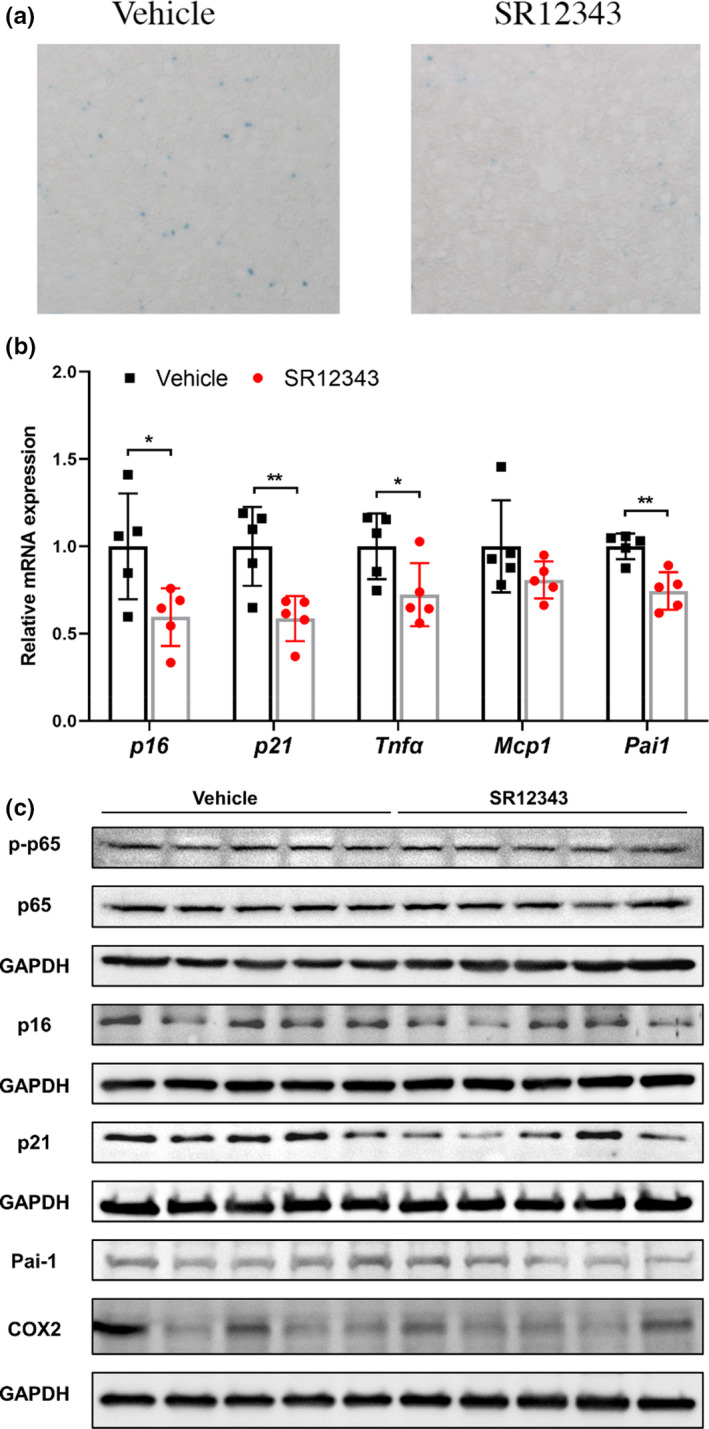
Chronic treatment of SR12343 reduces cellular senescence in liver of *Ercc1* ^−/^ *^Δ^* mice. (a) Liver sections of treated or untreated *Ercc1* ^−/^ *^Δ^* mice were stained SA‐β‐gal activity. Ten random fields at 10× magnification were captured and data were presented as the average of the absolute number of SA‐β‐gal^+^ cells. (b) RT‐qPCR analysis of liver tissues of *Ercc1* ^−/^ *^Δ^* mice was performed to evaluate the expression of senescent markers and SASP factors. Error bars indicate SEM. *n* = 5 per group. (c) Western blots showing expression of p‐p65/p65, p16, p21, Pai‐1, and COX2 in the liver of *Ercc1* ^−/^ *^Δ^* mice treated with vehicle or SR12343. *n* = 5 per group

We previously demonstrated that NF‐κB activation significantly increased in *Ercc1* ^−/^ *^Δ^* progeroid mice (Tilstra et al., 2012), and these mice have accelerated onset of senescence in different tissues due to DNA repair deficiency, yet in a pattern similar to WT mice (Yousefzadeh et al., 2020). To assess the effect of SR12343 on senescence in *Ercc1* ^−/^ *^Δ^* mice, X‐gal staining for senescence‐associated beta‐galactosidase (SA‐β‐gal) activity was performed on liver sections to determine the senescent (SA‐β‐gal^+^) cells. There was a significant reduction in the number of SA‐β‐gal positive cells in the liver of SR12343‐treated animals (Figures 3a and S2b). RT‐qPCR analysis of liver showed reduced expression of senescence and SASP markers including *p16^Ink4a^*, *p21^Cip1^*, *Tnfα*, and *Pai1* (Figure 3b). In addition, immunoblotting confirmed that SR12343 treatment reduced expression of p16^INK4a^, p21^CIP1^, Pai‐1 as well as NF‐κB activation at the protein level (Figures 3c and S2c).

### 2.4 SR12343 reduces markers of senescence in multiple tissues and attenuates muscle pathologies of *Ercc1* ^−/Δ^ progeroid mice

**FIGURE 4:**
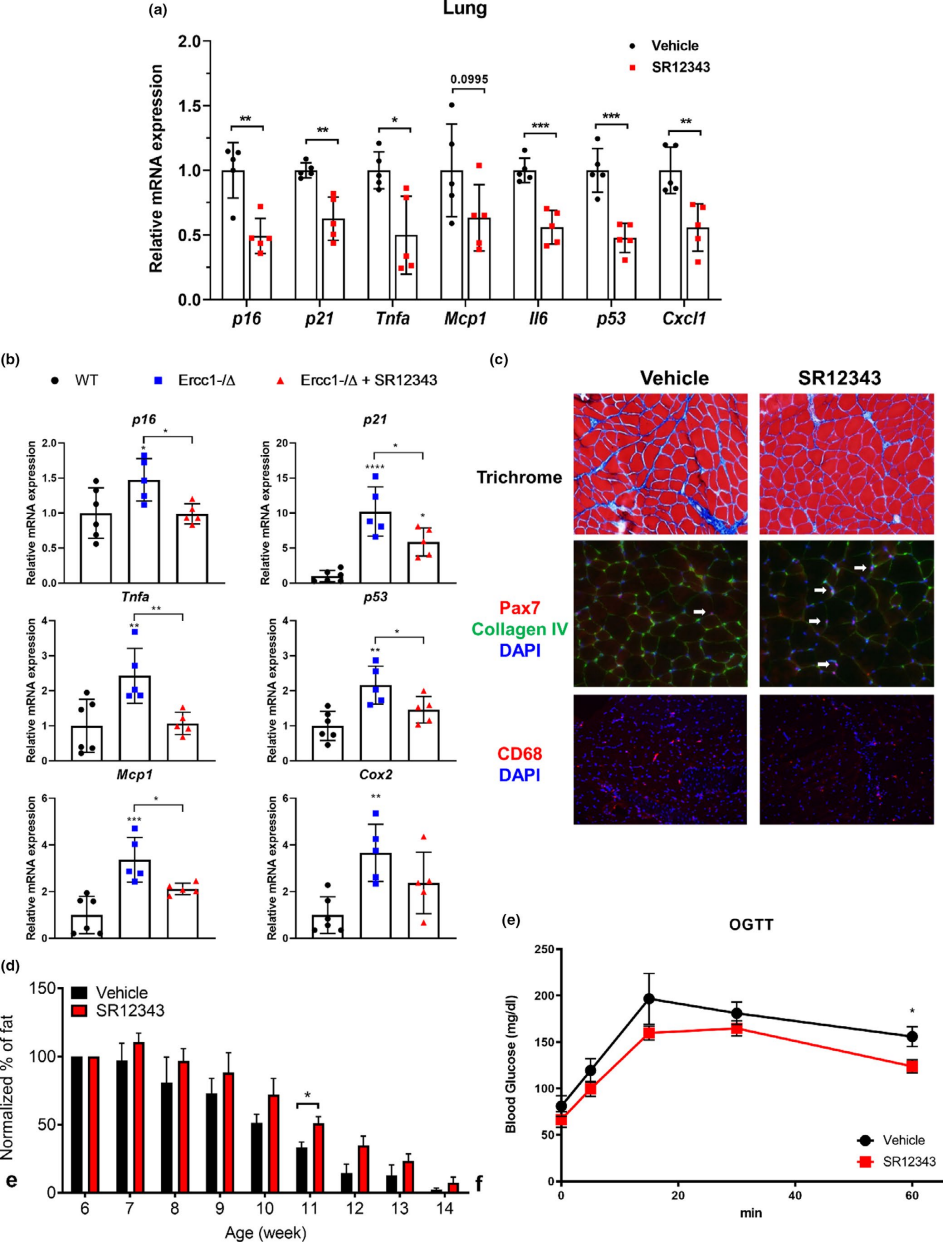
Chronic treatment of SR12343 reduces cellular senescence in multiple tissues and improves muscle pathologies of *Ercc1* ^−/^ *^Δ^* mice. RT‐qPCR analysis of (a) lung and (b) quadriceps of *Ercc1* ^−/^ *^Δ^* mice was performed to evaluate the expression of senescent markers and SASP factors. Error bars indicate SEM. *n* = 5 per group. (c) Skeletal muscles of SR12343 treated or untreated mice from 6 to 14 weeks of age were stained to compare the progression of fibrosis formation (trichrome staining), the number of Pax7^+^ muscle stem cells (Collagen Type IV was co‐stained to localize myofibers), and the number of CD68^+^ macrophages. (d) NMR was performed weekly on SR12343 treated or untreated mice from 6 to 14 weeks of age. Percentage of fat in males is shown and was normalized to the level at 6 weeks of age. Error bars indicate SE. *n* = 3–5 each group. (e) *Ercc1* ^−/^ *^Δ^* mice fasted overnight were dosed with a bolus of glucose (2g/kg) by oral gavage and blood glucose was measured at 0, 5, 15, 30 and 60 min. Glucose area under the curve (AUC) were measured. Error bars indicate SEM. *n* = 3–4 each group

The effect of SR12343 on senescence was also analyzed in the muscle of *Ercc1* ^−/^ *^Δ^* progeroid mice. With chronic treatment of S12343, there is a significant reduction of several senescence and SASP factors at the transcription level in the lung (Figure 4a) and quadriceps (Figure 4b), for example *p16^Ink4a^*, *p21^Cip1^*, *Tnfα*, *p53*, and *Mcp1*. The Masson trichrome staining procedure, where the collagen‐rich fibrotic regions stain blue, was used to assess the extent of fibrosis in skeletal muscle. In the SR12343‐treated muscles, the regions and extent of fibrosis were significantly reduced (Figures 4c and S2d). In addition, SR12343 treatment increased the number of Pax7^+^ satellite cells, consistent with improved muscle regeneration (Figures 4c and S2d). Moreover, a decreased number of CD68^+^ macrophages was observed, suggesting reduced inflammatory cell infiltration (Figures 4c and S2d).

### 2.5 SR12343 delays lipodystrophy and improves metabolic function in *Ercc1* ^−/Δ^ mice

Multiple progeria mouse models, such as *Zmpste24* ^−/−^, *Ercc1* ^−/−^, and aP2‐cre; *Ercc1^fl^* ^/−^, have been shown to exhibit reduced fat depots similar to naturally aged WT mice (Karakasilioti et al., 2013; Peinado et al., 2011). We employed nuclear magnetic resonance (NMR) to determine body composition, including fat mass and lean tissue mass in *Ercc1* ^−/^ *^Δ^* mice (Tinsley et al., 2004). There was a gradual decline of fat mass with aging starting from 8 weeks, which paralleled the onset of aging symptoms (Figures 4d and S2a). In particular, severe loss of fat was observed around 14 weeks of age, at which point relative fat mass in *Ercc1* ^−/^ *^Δ^* mice approached 0% (Figures 4d and S2a). However, fat loss was mitigated in SR12343 treated mice, suggesting that suppression of NF‐κΒ attenuates the progressive loss of this critical body organ (Figure 4d).

An association of impaired glucose tolerance with reduced body‐fat mass has been established (El‐Haschimi et al., 2003; Gavrilova et al., 2000; Shimomura et al., 1999). To determine if SR12343 affects metabolism, an oral glucose tolerance test (OGTT) was conducted on mice fasted overnight. The SR12343‐treated mice had lower plasma glucose levels at all time points and a smaller area under the curve (AUC), indicating accelerated glucose clearance and better insulin sensitivity (Figures 4e and S2e). To determine whether the levels of cellular senescence were altered in adipose tissue, SA‐β‐gal staining of parametrial fat showed reduced staining in treated mice, consistent with less senescence in fat (Figure S2f).

### 2.6 SR12343 reduces markers of senescence and improves muscle pathologies in *Zmpste24* ^−/−^ progeroid mice

We further investigated the therapeutic effects of SR12343 on *Zmpste24*‐knockout mice, a model of HGPS and an established segmental model of premature aging (Osorio et al., 2009). ZMPSTE24 is a zinc metalloproteinase that catalyzes the maturation of prelamin A to lamin A, an essential structural component of the nuclear envelope. *Zmpste24* deficiency leads to accumulation of unprocessed prelamin A within the nuclear envelope, resulting in nuclear architecture abnormalities, increased DNA damage, and shortened lifespan (Bergo et al., 2002). This accumulation of prelamin A drives many features of premature aging, especially the physiological and phenotypical changes in the musculoskeletal system, in *Zmpste24* ^−/−^ mice (Pendas et al., 2002). Importantly, *Zmpste24*‐deficient mice have constitutive NF‐κB hyperactivation in multiple tissues (Osorio et al., 2012).

**FIGURE 5:**
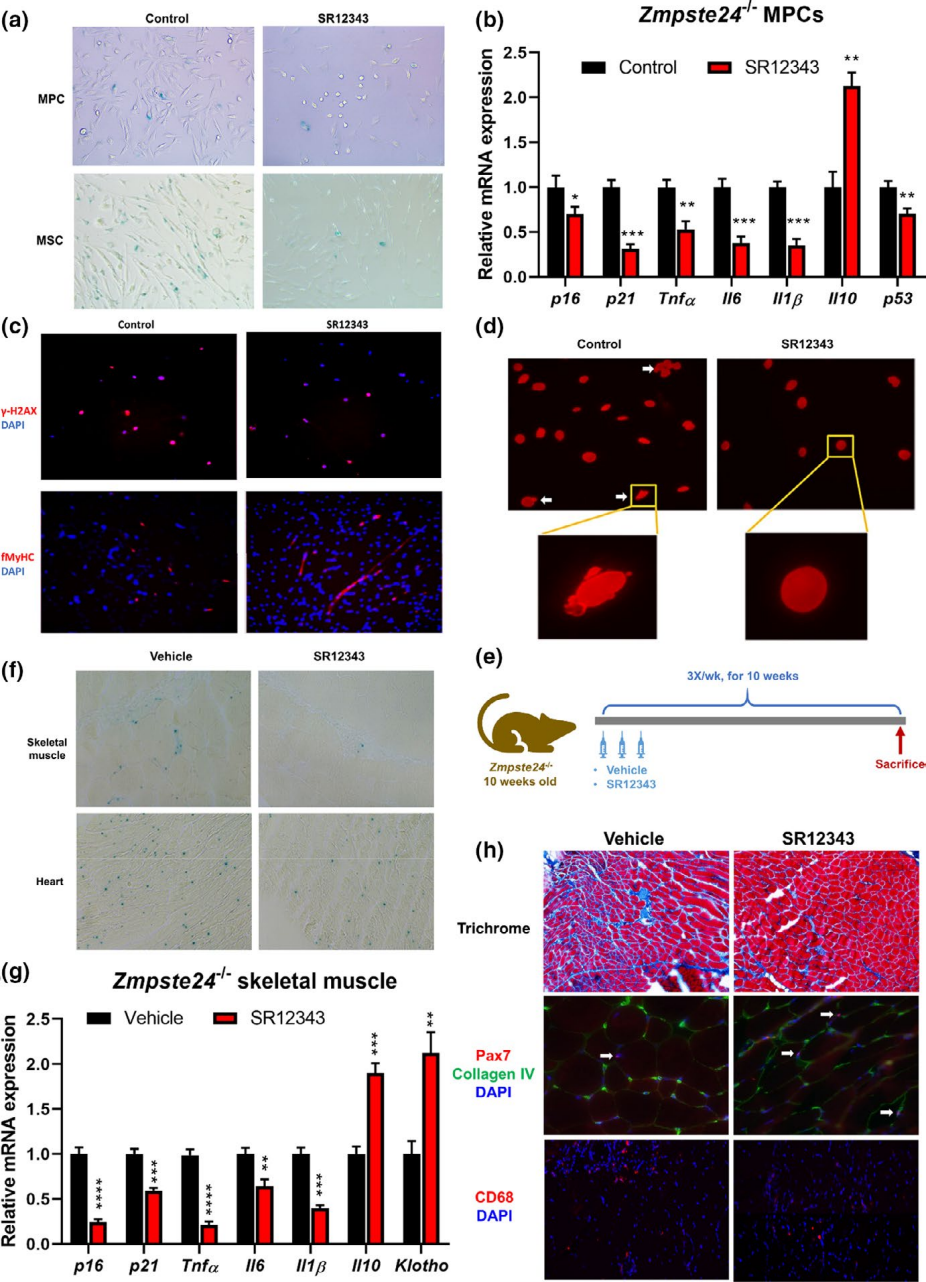
SR12343 reduces cellular senescence and improves muscle pathologies in the context of *Zmpste24* ^−/−^. (a) *Zmpste24* ^−/−^ MPCs and MSCs were treated with or without SR12343 (50 µM) for 4 days and the number of SA‐β‐Gal^+^ senescent cells evaluated. (b) RT‐qPCR analysis of the expression of genes associated with senescence and SASPs in *Zmpste24* ^−/−^ MPCs with or without SR12343 treatment (50 µM) for 2 days. Error bars indicate SEM for *n* = 3. (c) Immunofluorescence staining of γH2AX and FMyHC in *Zmpste24* ^−/−^ MPCs with or without SR12343 treatment (50 µM for 4 days). (d) Immunofluorescence staining of lamin A/C in *Zmpste24* ^−/−^ MPCs with or without SR12343 treatment (50 µM for 2 days), to track the occurrence of nuclear blebbing. (e) *Zmpste24* ^−/−^ mice at 10 weeks old were treated intraperitoneally with 30 mg/kg of SR12343 or vehicle three times per week for 10 weeks. After treatment, mice were sacrificed and tissues collected for analysis. (f) Representative images of SA‐β‐Gal staining for senescent cells were performed in skeletal muscle and cardiac muscles of *Zmpste24* ^−/−^ mice with or without SR12343 treatment. (g) RT‐qPCR analysis of the expression of genes associated with senescence and SASPs in the skeletal muscles (gastrocnemius) of *Zmpste24* ^−/−^ mice with or without SR12343 treatment. Error bars indicate SEM for *n* = 3. (h) Skeletal muscles (gastrocnemius) of SR12343 treated or untreated mice were analyzed to compare the progression of fibrosis formation (trichrome staining), the number of Pax7^+^ muscle stem cells (Collagen Type IV was co‐stained to localize myofibers), and the number of CD68^+^ macrophages

Muscle progenitor cells (MPCs) isolated from *Zmpste24* ^−/−^ mice exhibit increased SA‐β‐gal activity and impaired myogenic differentiation capacity compared to WT MPCs (Kawakami et al., 2019). *Zmpste24* ^−/−^ MPCs were cultured with or without SR12343 and maintained for 4 days prior to staining for SA‐β‐gal activity. The extent of cellular senescence in *Zmpste24* ^−/−^ MPCs was decreased as evidenced by the reduced number of SA‐β‐gal^+^ senescent cells. (Figures 5a and S3a). A similar reduction of senescence markers was seen in non‐myogenic mesenchymal stem cells (MSCs) following SR12343 treatment (Figures 5a and S3a). Furthermore, RT‐qPCR analysis of MPCs demonstrated that SR12343 treatment markedly reduced the expression of pro‐inflammatory SASP and senescence genes such as *p16^Ink4a^*, *p21^Cip1^*, *Tnfα*, *Il6*, *Il1β*, and *p53* while up‐regulating the anti‐inflammatory gene *Il10* (Figure 5b). Immunostaining of SR12343‐treated MPCs with an anti‐γH2AX antibody, a marker for DNA damage and senescence, revealed fewer γH2AX^+^ cells (Figures 5c and S3b).

*Zmpste24* ^−/−^ MPCs display limited myogenic differentiation capacity (Song et al., 2013). To test whether SR12343 improves the impaired myogenesis of *Zmpste24* ^−/−^ MPCs, the myogenic potential of MPCs was quantitated by immunostaining for the terminal myogenic differentiation marker of the fast‐type myosin heavy chain (f‐MyHC). With SR12343 treatment, *Zmpste24* ^−/−^ MPCs formed significantly more elongated multinucleated myotubes expressing f‐MyHC, indicating improved myogenic differentiation compared to untreated control (Figures 5c and S3b). *Zmpste24* deficiency and accumulation of prelamin A leads to nuclear blebbing. However, treatment with SR12343 resulted in improved nuclear architecture with reduced blebbing (Figures 5d and S3c).

The *in vivo* effect of 10 weeks of SR12343 treatment (30 mg/kg 3 times per week i.p.) was examined in the *Zmpste24* ^−/−^ mice starting at 10 weeks of age (Figure 5e). SR12343 treatment significantly increased the body weights of the *Zmpste24* ^−/−^ mice compared to the vehicle group (Figure S3e). The number of SA‐β‐gal positive cells in the skeletal muscle and heart tissues of *Zmpste24* ^−/−^ mice was significantly decreased compared to non‐treated mice (Figures 5f and S3d). Moreover, SR12343 treatment down‐regulated senescence and SASP genes (e.g., *p16^Ink4a^*, *p21^Cip1^*, *Tnfα*, *Il6* and *Il1β*) and up‐regulated the anti‐inflammatory gene *Il10* and longevity gene *Klotho* in skeletal muscle of *Zmpste24* ^−/−^ mice (Figure 5g). Consistent with the results in *Ercc1* ^−/Δ^ mice (Figure 4c), SR12343 treatment improved multiple muscle pathologies in *Zmpste24* ^−/−^ mice (Figures 5h and S3f). Specifically, trichrome staining of skeletal muscle tissues showed that fibrotic tissue (collagen type 1 positive) was reduced in SR12343‐treated muscles. Also, immunostaining of the muscle tissues revealed that the number of Pax7^+^ muscle satellite cells was increased, consistent with improved muscle regeneration potential. Also, the number of CD68^+^ macrophages in muscle was decreased after SR12343 treatment, suggesting less inflammatory cell infiltration.

### 2.7 Late‐life SR12343 treatment improved multiple health conditions in naturally aging mice

**FIGURE 6:**
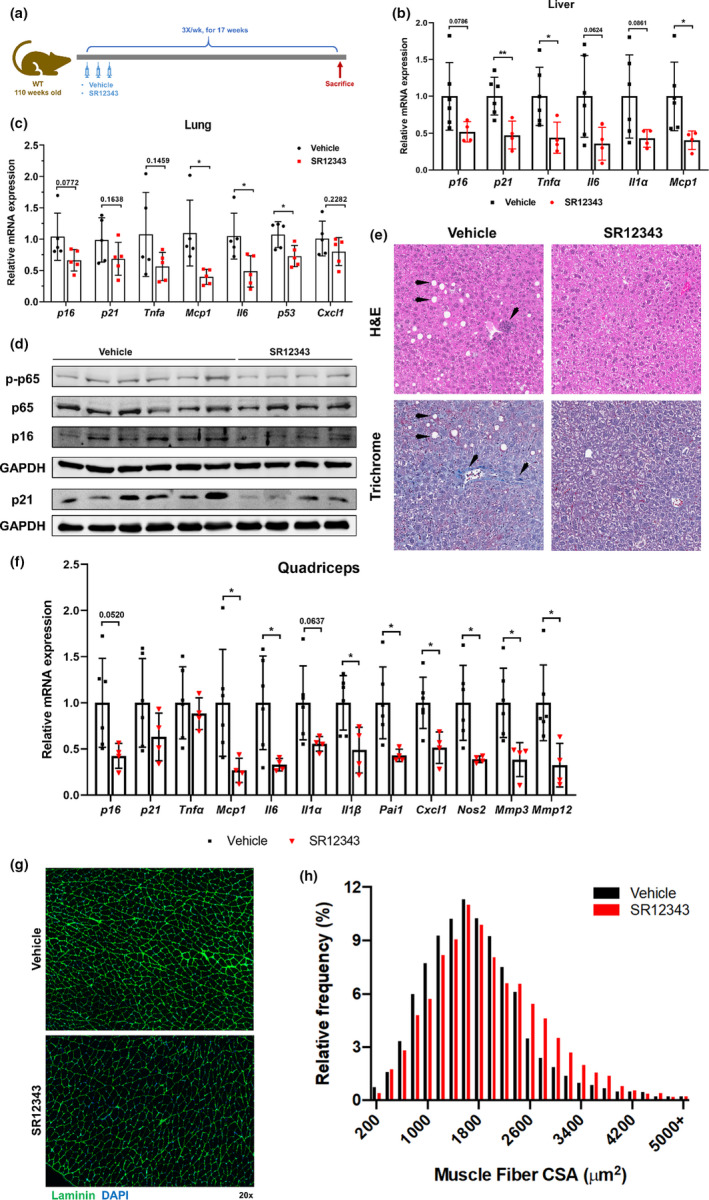
Chronic treatment of SR12343 reduces cellular senescence and improves pathologies in multiple tissues of old WT mice. (a) Wild‐type mice at 110 weeks old were treated intraperitoneally with vehicle or 30 mg/kg of SR12343 for 17 weeks before sacrifice for tissue analysis. RT‐qPCR analysis of the (b) liver and (c) lung of wild‐type old mice was performed to evaluate the expression of senescent markers and SASP factors. Error bars indicate SEM. Vehicle, *n* = 6; SR12343, *n* = 4. (d) Western blots showing expression of p‐p65/p65, p16, and p21 in the liver of wild‐type old mice treated with vehicle or SR12343. (e) Representative images of H&E and Masson's trichrome staining of liver sections of vehicle and SR12343 treated naturally aged mice. Images were taken at 10×. (f) RT‐qPCR analysis of quadriceps of wild‐type old mice was performed to evaluate the expression of senescent markers and SASP factors. Error bars indicate SEM. Vehicle, *n* = 6; SR12343, *n* = 4. (g) Representative images of immunofluorescence for laminin of muscle sections in vehicle and SR12343 treated naturally aged mice. (h) Distribution of cross‐sectional areas (CSAs) of muscle fibers. Error bars indicate SEM. *n* = 3 per group.

Lastly, the effect of SR12343 on healthspan was investigated in wild‐type old mice as a late life intervention. Wild‐type C57BL/6J:FVB/NJ mice (~110 weeks old) were treated with SR12343 (30 mg/kg) three times per week intraperitoneally for 17 weeks (Figure 6a). RT‐qPCR analysis of the liver (Figure 6b) and lung (Figure 6c) tissues showed that multiple senescence markers and SASP genes were either significantly reduced or were trending toward significance compared to vehicle treated mice, including *p21^Cip1^*, *Tnfα*, and *Mcp1*. Consistently, chronic treatment of SR12343 also reduced the level of senescence markers p16^INK4a^ and p21^CIP1^ in the liver at the protein level as determined by immunoblotting (Figures 6d and S3h). Similarly, despite the fact that the mice were only treated 3 times per week with SR12343, the level of phospho‐p65 in the liver was reduced. Furthermore, histological analysis by H&E and trichrome staining found that SR12343 treatment reduced the fat accumulation (steatosis), deposits of collagen fibers (fibrosis) and tissue scars (cirrhosis) in liver of WT old mice (Figure 6e). These results are consistent with SR12343 attenuating liver pathologies by reducing markers of cellular senescence.

The muscle tissue of the aged, SR12343‐treated WT mice was also examined. SR12343 treatment resulted in significant reduction of senescence and SASP markers at the transcription level, including *p16^Ink4a^*, *p21^Cip1^*, *Il6*, *Mcp1* and *Pai1*. (Figure 6f). Furthermore, histological analysis of skeletal muscle cross sections revealed an increase in the average size of fibers and percentage of larger muscle fibers (Figures 6g,h and S3i), indicating attenuation of muscle atrophy associated with aging. These results suggest that pharmacologically inhibiting IKK/NF‐κB hyperactivation with SR12343 can reduce markers of senescence in muscle and attenuate muscle atrophy in aged mice.

## 3 DISCUSSION

The relationships between aging and NF‐κB have been demonstrated in a plethora of *in vitro* and *in vivo* studies showing that NF‐κB is hyperactivated with age, driving inflammation, stem cell dysfunction and age‐related diseases and pathologies. Importantly, chronic inflammation driven, in part, by NF‐κB regulated genes is one of the hallmarks of aging (Balistreri et al., 2013; Lopez‐Otin et al., 2013). Most inducers of senescence and aging, such as genotoxic, oxidative and inflammatory stresses, also are triggers of NF‐κB signaling. This activation of NF‐κB by stress can then induce expression of certain SASP genes, for example, *Il1α*, *Il1β*, *Il6*, *Il8* and *Tnfα*. Recently, in an effort to discover rare protective variants in genes implicated in healthy aging, an individual target capture sequencing (Capture‐Seq) was performed to discover all the possible variants in 568 candidate genes in 51 centenarians and 51 controls with gene‐based association analysis. Non‐coding rare variants were identified in centenarians in three different NF‐κB family members, *RELA*, *NFKB1*, and *NFKBIA* (Ryu et al., 2021). These rare variants appear to reduce canonical NF‐κB activity. Thus, reducing NF‐κB activity later in life may provide a therapeutic strategy to slow aging. However, since reducing the basal level of NF‐κB can have adverse effects on cell survival, targeting IKK to reduce the extent of NF‐κB activation without affecting basal activity represents a safer therapeutic approach. Previous experiments using a small peptide derived from the region of IKKβ that binds directly to NEMO, termed the NEMO Binding Domain (NBD), have demonstrated the therapeutic benefits of targeting IKK for treatment of a variety of pre‐clinical disease models including muscular dystrophy (Reay et al., 2011), Parkinson's disease (Ghosh et al., 2007), arthritis (Dai et al., 2004), senescence and aging (Tilstra et al., 2014). Therefore, we focused our efforts on developing a more clinically relevant and effective small molecule inhibitor of IKK, targeting the interaction between IKKβ and NEMO similarly as the NBD peptide, as a potential therapy for reducing senescence, SASP and fibrosis while improving stem cell function and extending healthspan (Salminen & Kaarniranta, 2009; Tilstra et al., 2011).

The hyperactivation of NF‐κB has been observed in various murine models of progeroid and normal aging, including *Sirt6* ^−/−^, *Ercc1* ^−/^ *^Δ^* and *Zmpste24* ^−/−^ progeroid mice (Kawahara et al., 2009; Osorio et al., 2012; Tilstra et al., 2012). Previous reports by our group and others have shown that genetic inhibition of NF‐κB hyperactivation reduces cellular senescence in tissues and leads to improved pathologies and extended life span (Adler et al., 2007; Kawahara et al., 2009; Osorio et al., 2012; Tilstra et al., 2012). However, studies based on chronic pharmacological inhibition of IKK/NF‐κB by specific compounds showing an extension of healthspan are still limited. In this study, we demonstrate that inhibiting IKK activation of NF‐κB with SR12343, a novel small molecule that specifically disrupts the association between NEMO and IKKβ (Zhao et al., 2018), reduced senescence and SASP factors in multiple tissues, reduced fibrosis and cellular infiltration while increasing the number of satellite cells in muscle and improved healthspan in multiple tissues of both progeroid and naturally aging mice. These findings not only reinforce a causal role of IKK/NF‐κB hyperactivation in contributing to driving aging and age‐related diseases, but also support the use of SR12343‐like inhibitors of IKK/NF‐κB for extending healthspan.

In all the mouse models of aging, one striking therapeutic effect of inhibiting IKK/NF‐κB hyperactivation by SR12343 was the improvement in muscle pathologies including reduced fibrosis, increased number of Pax7^+^ satellite cells and reduced inflammatory infiltration. There was also an increase in muscle fiber size in old WT and *Ercc1* ^−/∆^ mice, consistent with a reduction in age‐related atrophy. In addition, SR12343 treatment consistently showed reduced markers of cellular senescence as evidenced by the reduction in SA‐β‐gal activity and expression of senescence and SASP genes in muscle tissue. The reduction of senescence markers also was observed in both mouse and human senescent cells in culture induced by either genotoxic or oxidative stress. These results suggest that the improved muscle pathologies are mediated, at least in part, by the reduction of senescence markers and the attenuation of cell non‐autonomous pathways such as the SASP. In addition, there may be crosstalk between NF‐κB signaling with other pro‐aging and longevity pathways. For example, skeletal muscle myogenesis is tightly modulated by Wnt/β‐catenin signaling which regulates self‐renewal and the progression of muscle precursors along the myogenic lineage (Maltzahn et al., 2012). NF‐κB activation can inhibit the Wnt/β‐catenin pathway either indirectly through the functions of NF‐κB target genes or directly by interfering with the formation of transcriptional complex β‐catenin/TCF/p300 (Ma & Hottiger, 2016). Furthermore, we previously demonstrated that *p65* ^+/–^ muscle‐derived stem cells with genetically reduced NF‐κB activation showed increased capacity for myogenic regeneration after skeletal muscle implantation (Lu et al., 2012). Thus, the muscle pathologies improved by pharmacologic inhibition of IKK/NF‐κB could also be due to enhanced stem cell proliferation and muscle regeneration. Given our previous results showing a therapeutic effect of SR12343 on dystrophic muscle in *mdx* mice, these results strongly support the use of SR12343 or related compounds to slow muscle aging.

In addition to the effects of SR12343 on muscle pathology, multiple neurological symptoms in *Ercc1* ^−/^ *^Δ^* mice, such as dystonia, gait disorder, hindlimb paralysis and ataxia, were improved by SR12343. It is important to note that it is still not clear if the improvement on neurodegeneration is due to cell autonomous or non‐autonomous effects of IKK/NF‐κB inhibition in other tissues such as muscle. However, previous analysis of biodistribution showed that SR12343 indeed crosses the blood‐brain barrier (Zhao et al., 2018).

In summary, we demonstrate the therapeutic activity of chronic treatment with the IKK/NF‐κB inhibitor SR12343 in terms of reducing cellular senescence, extending healthspan, attenuating metabolic abnormality and improving tissue pathologies in murine models of premature aging as well as natural aging. Our results suggest that inhibiting the IKK‐mediated activation of NF‐κB signaling represents a promising target for the development of drug interventions for healthy aging. Moreover, SR12343 is a potential therapeutic compound warranting further development for anti‐aging interventions and treating age‐related diseases.

## 4 MATERIALS AND METHODS

### 4.1 Cells and mice

Primary *Ercc1* ^−/−^ mouse embryonic fibroblasts (MEFs) were isolated on embryonic day 12.5–13.5. In brief, mouse embryos were isolated from yolk sac followed by the complete removal of viscera, lung and heart if presented. Embryos were then minced into fine chunks, fed with MEFs medium, cultivated under 3% oxygen to reduce stresses. Cells were split at 1:3 when reaching confluence. MEFs were grown at a 1:1 ratio of Dulbecco's Modification of Eagles Medium (supplemented with 4.5 g/L glucose and L‐glutamine) and Ham's F10 medium, supplemented with 10% fetal bovine serum, penicillin, streptomycin and non‐essential amino acid. To induce oxidative stress‐mediated DNA damage, *Ercc1* ^−/−^ MEFs were switched to 20% oxygen for three passages.

Human IMR90 lung fibroblasts were obtained from American Type Culture Collection (ATCC) and cultured in EMEM medium with 10% FBS and pen/strep antibiotics. To induce senescence, cells were treated with 20 μM etoposide for 48 h, followed by 6 days in normal culture media.

*Ercc1* ^+/−^ and *Ercc1* ^+/^ *^Δ^* mice from C57BL/6J and FVB/n backgrounds were crossed to generate *Ercc1* ^−/^ *^Δ^* mice to prevent potential strain‐specific pathology. For drug treatment, 6–8‐week‐old *Ercc1* ^−/^ *^Δ^* mice were dosed with 30 mg/kg of SR12343 or vehicle by intraperitoneal injection three times per week up until 15 weeks of age. SR12343 was formulated in 10:10:80 of DMSO:Tween 80:water for in vivo administration. Animal protocols used in this study were approved by Scripps Florida and University of Minnesota Institutional Animal Care and Use Committees.

*Zmpste24* ^−/−^ mice (B6.129SZmpste24tm1Sgy/Mmucd) were studied as an established model for Hutchinson‐Gilford Progeria Syndrome (HGPS). The aged‐matched littermates (*Zmpste24* ^+/+^) mice born from same *Zmpste24* ^+/−^ parents were used as wild‐type controls. Both male and female mice were used for this study since both genders are susceptible to HGPS disease. All mice were housed and maintained in the Center for Laboratory Animal Medicine and Care (CLAMC) at UTHealth in accordance with established guidelines and protocols approved by the UTHealth Animal Welfare Committee.

Aged wild‐type C57BL/6J:FVB/NJ mice were generated by crossing C57BL/6J and FVB/n inbred mice purchased from Jackson Laboratory (Stock No.:000664 and 001800). Mice were left to age for 2 years before being enrolled into the late life intervention study. Once the cohort was of over 2 years, mice over 110 weeks were treated with 30 mg/kg of SR12343 or vehicle by intraperitoneal injection three times per week for 17 weeks. Animal protocols used in this study were approved by Scripps Florida and University of Minnesota Institutional Animal Care and Use Committees.

### 4.2 Health evaluation of *Ercc1* ^−/^ *^Δ^* mice

Health assessment of *Ercc1* ^−/^ *^Δ^* mice was conducted twice per week to evaluate age‐related symptoms, including body weight, tremor, forelimb grip strength, kyphosis, hindlimb paralysis, gait disorder, dystonia and ataxia. Kyphosis, body condition and coat condition were used to reflect general health conditions. Ataxia, dystonia, gait disorder and tremor were used as indicators of aging‐related neurodegeneration. Muscle wasting was studies by monitoring hindlimb paralysis and forelimb grip strength. All aging symptoms were scored based on a scale of 0, 0.5 and 1, with the exception of dystonia that has a scale from 0 to 5. The sum of aging scores of each group was used to determine the overall aging conditions, with zero means no symptom presented.

### 4.3 Nuclear magnetic resonance (NMR)

Bruker's minispec LF50 body composition analyzer was used to measure lean tissue, fat and fluid in mice according to the manufacturer's instructions. *Ercc1* ^−/^ *^Δ^* mice were placed into a restrainer and body composition was measured by the analyzer. Readings of lean tissue, fat and fluid were normalized to body weight to get the percentage of each body composition.

### 4.4 Oral glucose tolerance test (OGTT)

*Ercc1* ^−/^ *^Δ^* mice at 11‐weeks old were fasted overnight prior to OGTT test. Baseline fasting blood glucose was obtained before glucose administration at 0 min. A bolus of glucose (2 g/kg) was administrated to each mouse by oral gavage and blood glucose was measured by glucometer at 5, 15, 30, 60 min with tail‐tip blood.

### 4.5 SA‐β‐gal senescence assay by C12FDG staining

*Ercc1* ^−/−^ MEFs were passaged 3 times at 20% O_2_ to induce senescence then seeded at 2000 cells per well in black wall, clear bottom 96‐well plates at least 6 h prior to treatment. Following the addition of SR12343 or control, the *Ercc1* ^−/−^ MEFs were incubated for 48 h at 20% O_2_. After removing the medium, cells were incubated in 100 nM Bafilomycin A1 in culture medium for 1 h to induce lysosomal alkalinization, followed by incubation with 20 μM fluorogenic substrate C_12_FDG (Setareh Biotech) for 2 h and then counterstaining with 2 μg/ml Hoechst 33342 (Thermo Fisher) for 15 min. Subsequently, cells were washed with PBS and fixed in 2% paraformaldehyde for 15 min. Finally, cells were imaged with six fields per well using a high content fluorescent image acquisition and analysis platform Cytation 1 (BioTek).

### 4.6 EdU cell proliferation assay

After drug treatment, cells were fixed and stained using a Click‐iT™ EdU Alexa Fluor 594 Assay Kit (Thermo Fisher) according to the manufacturer's instructions.

### 4.7 X‐gal staining *in vitro*

Cellular senescence was evaluated in cultured cells using the senescence β‐galactosidase staining kit (Cell Signaling Technology) for SA‐β‐gal activity, according to the manufacturer's protocol. Images were obtained and evaluated using a bright‐field microscopy. The number of cells that were SA‐β‐gal positive was calculated and averaged across 15 fields, each from three replicate plates in four independent experiments. The percentage of senescent cells was calculated as SA‐β‐gal^+^ cells divided by the total number of cells and then multiplied by 100.

### 4.8 X‐gal staining *ex vivo*

Fresh fat tissues of *Ercc1* ^−/^ *^Δ^* mice were fixed in 2% formaldehyde and 0.2% glutaraldehyde in PBS for 10 min at room temperature. After the removal of fixative, adipose tissue was stained with SA‐β‐gal staining (pH 6.0) solution (40 mM citric acid in sodium phosphate buffer, 5 mM K_4_[Fe(CN)_6_] _3_H2O, 5 mM K_3_[Fe(CN)_6_], 150 mM sodium chloride, 2 mM magnesium chloride and 1 mg/ml X‐gal dissolved in N,N‐dimethylformamide) for 16–20 h in a 37℃ incubator without CO_2_ injection. Images were taken at 5 h into the staining.

Fresh liver tissue of *Ercc1* ^−/^ *^Δ^* mice was fixed in 10% neutral buffered formalin (NBF) for 3–4 h and then transferred to 30% sucrose overnight. Tissue was then imbedded in optimal cutting temperature compound (OCT) and stored at −80℃. Five μm cryosections were performed and slides were stained in SA‐β‐gal staining solution (pH 5.8) at 37℃ for 16–24 h. To quantify, 10 random images were captured using a bright‐field microscopy at 10× magnification and the number of SA‐β‐gal^+^ cells per field was counted.

Freshly frozen skeletal muscle and cardiac muscle tissues of *Zmpste24* ^−/−^ mice were cryosectioned to get 10 µm sections. The number of senescent muscle cells in muscle tissues were evaluated using the SA‐β‐gal staining kit (Cell Signaling) following the manufacturer's protocol.

### 4.9 RT‐qPCR analysis

Total RNA was extracted from cells or snap frozen tissues using Trizol reagent (Thermo Fisher). cDNA was synthesized using High‐Capacity cDNA Reverse Transcription Kit (Thermo Fisher). Quantitative PCR reactions were performed with FastStart Universal SYBR Green Master (Rox) from Roche. The experiments were performed according to the manufacturer's instructions. The sequences of the primers used were listed in Table S1.

### 4.10 Western blot analysis

Snap frozen tissues from *Ercc1* ^−/^ *^Δ^* and WT mice were lysed in RIPA lysis and extraction buffer (Thermo Fisher). Protein concentration was determined using BCA protein assay kit (Thermo Fisher). Equal amounts of protein were loaded onto SDS‐PAGE polyacrylamide gels then transferred to 0.2 µm pore size nitrocellulose membranes (Bio‐Rad). Membranes were blocked with 5% BSA in PBS‐Tween for 1 h at room temperature then incubated with primary antibodies overnight at 4℃. Membranes were then probed with secondary antibodies at room temperature. Protein expression was measured by fluorescence using iBright™ FL1000 Imaging System. The density of each blot was quantified by using ImageJ (NIH) normalized to GAPDH. The following primary antibodies were used in this study: rabbit anti‐GAPDH (Cell Signaling Technology, 2118), rabbit anti‐p16 (Santa Cruz, sc‐1207), rabbit anti‐p21 (Abcam, ab7960), rabbit anti‐p‐p65 (Cell Signaling Technology, 3033), mouse anti‐p‐p65 (Cell Signaling Technology, 6956), rabbit anti‐Pai‐1 (Santa Cruz, sc‐8979), rabbit anti‐COX2 (Cell Signaling Technology, 12282). The following secondary antibodies were used in this study: goat anti‐rabbit IgG (H+L) Alexa Fluor Plus 488 (Thermo Fisher, A32731), goat anti‐mouse IgG (H+L) Alexa Fluor 633 (Thermo Fisher, A21052).

### 4.11 Immunofluorescence

Cultured muscle cells were fixed with 4% paraformaldehyde. The primary antibodies for γH2AX (Cell Signaling), fast‐type Myosin Heavy Chain (FMyHC) (Abcam), and Lamin A/C (Santa Cruz) were used at a 1:100 to 1:300 dilution. The cells were analyzed via fluorescence microscopy (Nikon Instruments Inc. *Melville*, NY) and photographed at 4–40× magnification.

### 4.12 Muscle pathologies

Muscle tissues cryosections (10 µm) from *Ercc1* ^−/^ *^Δ^* and *Zmpste24* ^−/−^ mice were fixed with 10% formalin for 10 min. The primary antibodies for Pax7 (DHSB) and CD68 (Abcam) were used at a 1:100 to 1:300 dilution. All slides were analyzed via fluorescence microscopy (Nikon Instruments Inc.) and photographed at 4–40× magnification. The cell nuclei were stained with DAPI. Fibrosis formation in muscle tissues was visualized by Masson trichrome staining with the Trichrome Stain (Masson) Kit (Sigma‐Aldrich). Sections were incubated in Weigert's iron hematoxylin working solution for 10 min, and rinsed under running water for 10 min. Slides were transferred to Biebrich scarlet‐acid fuchsin solution for 15 min before incubation in aniline blue solution for another 5 min. Slides were then rinsed, dehydrated, and mounted as earlier. The ratio of the area of fibrotic collagen (blue) to the area of normal muscle (red) was quantified to measure fibrosis formation.

Skeletal muscle tissues of WT mice were embedded in OCT compound (Sakura Finetech USA Inc) and frozen in liquid nitrogen‐cooled 2‐methylbutane. Muscle sections (7 μm) were cut and then stained with anti‐laminin antibody (catalog number L9393, Sigma‐Aldrich). Images were acquired using a fluorescence microscope (Nikon Eclipse Ti, Nikon Instruments, Inc.) and the cross‐sectional area of individual muscle fibers was quantified using MuscleJ (Fiji software, NIH).

### 4.13 Statistical analysis

Data were statistically analyzed by Graphpad Prism software. Two‐tailed Student's *t*‐test was performed to determine differences between two groups, and one‐way ANOVA with Tukey's test was used for three groups. A value of *p* < 0.05 was considered as statistically significant, shown as *p < 0.05, **p < 0.01, ***p < 0.001 and ****p < 0.0001.

## CONFLICT OF INTEREST

PDR, LJN and TMK hold a patent on SR12343.

## AUTHOR CONTRIBUTIONS

The project was conceived by PDR and LJN. LZ and JZ performed the cell culture experiments and LZ, JZ and MJY performed the analysis of senescence and SASP in WT and *Ercc1* ^−/^ *^∆^* mice. SJM, LAA, and RDO bred and genotyped the *Ercc1* ^−/^ *^∆^* mice and performed the health assessments on Ercc1^−/∆^ and WT mice. TMK performed the synthesis of SR12343. XM performed all the studies in *Zmste24* ^−/−^ mice in the laboratory of JH. AS, ZA and NKL performed the analysis of muscle in *Ercc1* ^−/^ *^∆^* and WT mice. LZ, JZ, NKL, JH, TKM, YS, LJN and PDR designed the experiments and contributed to the writing of the manuscript. All authors read, edited, and approved the final version of the manuscript.

## Supporting information

Supplementary Material

Supplementary Material

## Data Availability

All data needed to evaluate the conclusions in the paper are present in the paper and/or the Supplementary Materials.
