# Supplementary material for: Novel small molecule inhibition of IKK/NF‐κB activation reduces markers of senescence and improves healthspan in mouse models of aging
Source: Aging Cell. 2021 Nov 3;20(12):e13486. doi: 10.1111/acel.13486 (PMC8672781; doi:10.1111/acel.13486)
Supplement: Supplementary file 1 — Supplementary Material [file ACEL-20-e13486-s001.docx]

**Supplementary Materials**

**Figure S1**.


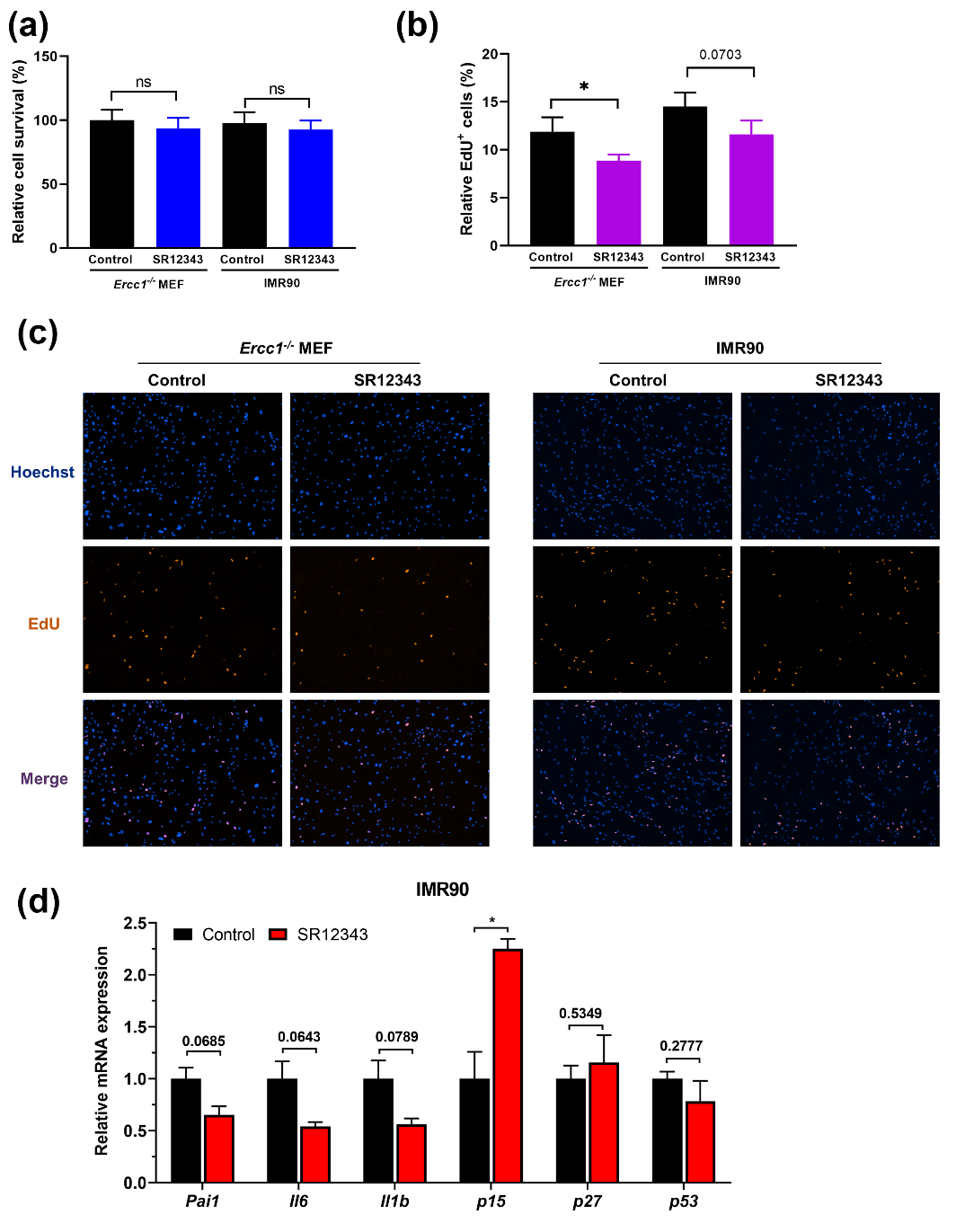


**Figure S2**.


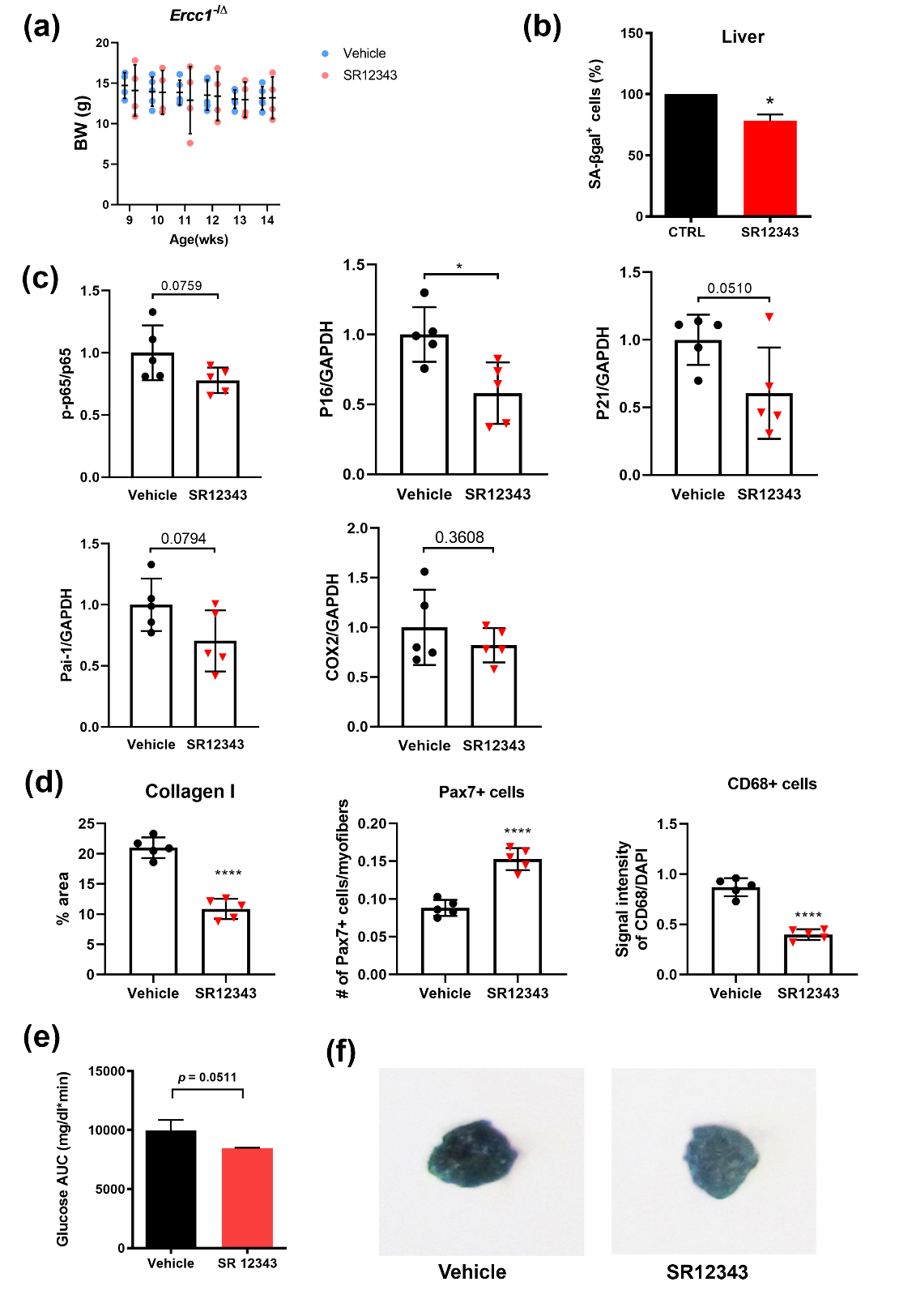


**Figure S3**.


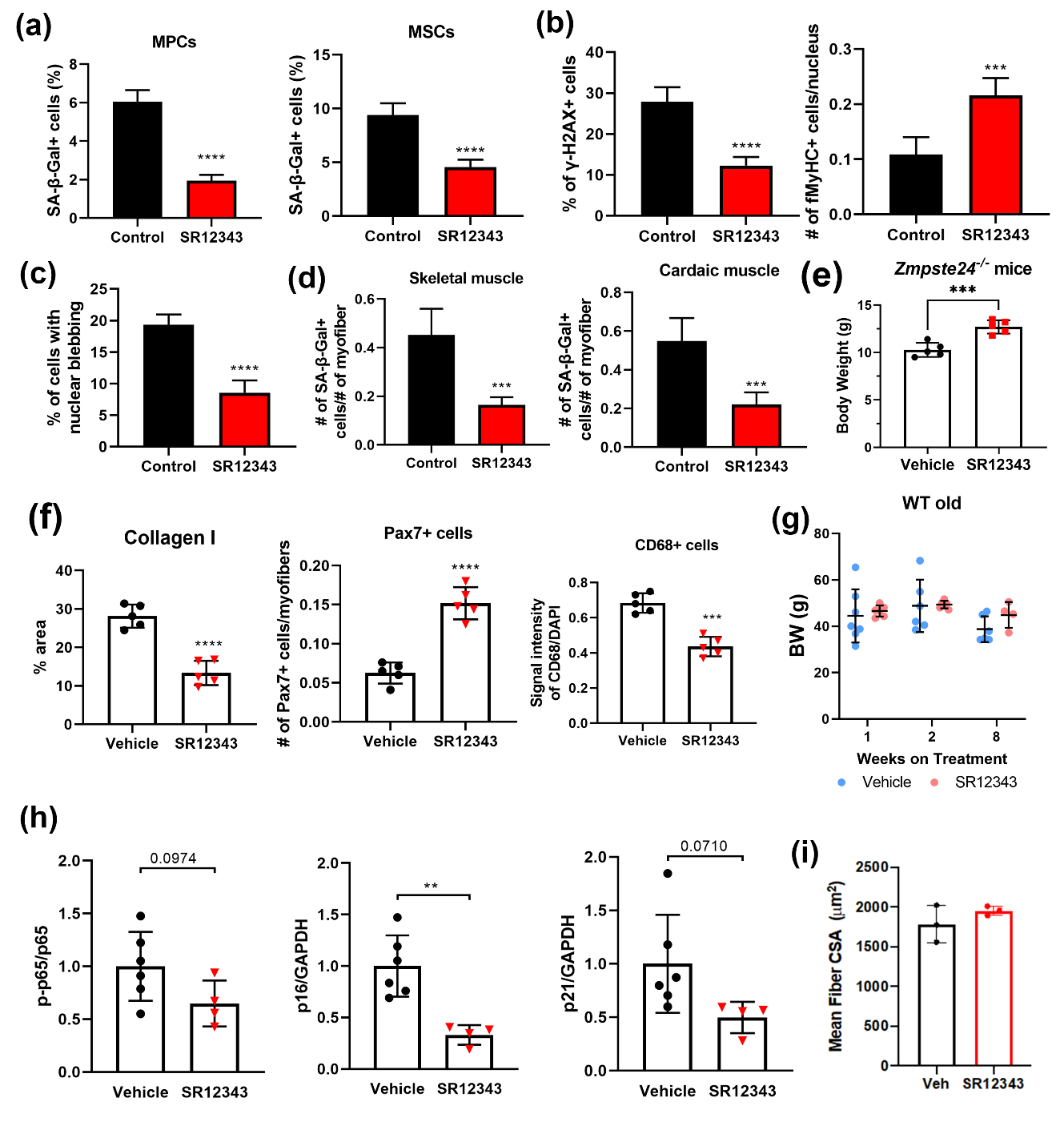


**Table S1**. List of primers used in this study.

| For *Ercc1^-/-^* MEFs, *Ercc1^-/Δ^* and WT mice | | |
| --- | --- | --- |
| Gene name | Forward sequence | Reverse sequence |
| *p21^Cip1^* | GTCAGGCTGGTCTGCCTCCG | CGGTCCCGTGGACAGTGAGCAG |
| *p16 ^Ink4a^* | CCCAACGCCCCGAACT | GCAGAAGAGCTGCTACGTGAA |
| *Il6* | TACCACTTCACAAGTCGGAGGC | CTGCAAGTGCATCATCGTTGTTC |
| *Il1α* | ACGGCTGAGTTTCAGTGAGACC | CACTCTGGTAGGTGTAAGGTGC |
| *Il1β* | TGGACCTTCCAGGATGAGGACA | GTTCATCTCGGAGCCTGTAGTG |
| *Tnfα* | GGTGCCTATGTCTCAGCCTCTT | GCCATAGAACTGATGAGAGGGAG |
| *Mcp1* | GCTACAAGAGGATCACCAGCAG | GTCTGGACCCATTCCTTCTTGG |
| *Pai1* | CCTCTTCCACAAGTCTGATGGC | GCAGTTCCACAACGTCATACTCG |
| *p53* | CTCTCCCCCGCAAAAGAAAAA | CGGAACATCTCGAAGCGTTTA |
| *Cox2* | GCGACATACTCAAGCAGGAGCA | AGTGGTAACCGCTCAGGTGTTG |
| *Cxcl1* | TCCAGAGCTTGAAGGTGTTGCC | AACCAAGGGAGCTTCAGGGTCA |
| *Nos2* | GAGACAGGGAAGTCTGAAGCAC | CCAGCAGTAGTTGCTCCTCTTC |
| *Mmp3* | CTCTGGAACCTGAGACATCACC | AGGAGTCCTGAGAGATTTGCGC |
| *Mmp12* | CACACTTCCCAGGAATCAAGCC | TTTGGTGACACGACGGAACAGG |
| *Gapdh* | AAGGTCATCCCAGAGCTGAA | CTGCTTCACCACCTTCTTGA |
| for *Zmpste24*^−/−^ cells and mice | | |
| Gene name | Forward Sequence | Reverse Sequence |
| *p16 ^Ink4a^* | AACTCTTTCGGTCGTACCCC | GCGTGCTTGAGCTGAAGCTA |
| *p21 ^Cip1^* | CCTGGTGATGTCCGACCTG | CCATGAGCGCATCGCAATC |
| *Tnfα* | CCTGTAGCCCACGTCGTAG | GGGAGTAGACAAGGTACAACCC |
| *Il6* | CTGCAAGAGACTTCCATCCAG | AGTGGTATAGACAGGTCTGTTGG |
| *Il1β* | GCAACTGTTCCTGAACTCAACT | ATCTTTTGGGGTCCGTCAACT |
| *Il10* | ATTTGAATTCCCTGGGTGAGAAG | CACAGGGGAGAAATCGATGACA |
| *Klotho* | ACTACGTTCAAGTGGACACTACT | GATGGCAGAGAAATCAACACAGT |
| *p53* | CCCCTGTCATCTTTTGTCCCT | AGCTGGCAGAATAGCTTATTGAG |
