# Supplementary material for: Novel small molecule inhibition of IKK/NF‐κB activation reduces markers of senescence and improves healthspan in mouse models of aging
Source: Aging Cell. 2021 Nov 3;20(12):e13486. doi: 10.1111/acel.13486 (PMC8672781; doi:10.1111/acel.13486)
Supplement: Supplementary file 2 — Supplementary Material [file ACEL-20-e13486-s002.docx]

**Supplementary Figures**

**Figure S1:** (**a**) Quantification of the numbers of live *Ercc1*^-/-^ MEF and IMR90 cells staining with Hoechst 33324 to indicate cell survival. Error bars indicate SEM for n = 3. (**b**) Quantification of EdU positive *Ercc1*^-/-^ MEF and IMR90 cells. Error bars indicate SEM. n = 3. (**c**) Representative images of nuclear and EdU staining of *Ercc1*^-/-^ MEF and IMR90 cells with and without treatment of SR12343 (50 µM). (**d**) RT-qPCR analysis of expression of senescence biomarkers in senescent IMR90 cells with or without treatment of SR12343 (50 µM). Error bars indicate SEM for n = 4.

**Figure S2:** (**a**) Body weights of *Ercc1^-/Δ^* mice with or without SR12343 treatment. (**b**) Quantification of SA-β-gal staining of liver sections of *Ercc1^-/Δ^* mice. Error bars indicate SEM. Vehicle, n = 2; SR12343, n = 3. (**c**) Quantification of Western blotting in Fig. 3c. Error bars indicate SEM. n = 5 per group. (**d**) Quantification of trichrome staining for fibrosis, Pax7+ muscle stem cells, and CD68^+^ macrophages in skeletal muscles of *Ercc1^-/Δ^* mice in Fig. 4c. Error bars indicate SEM. n = 5 per group. (**e**) Quantification of glucose AUC in Fig. 4e. Error bars indicate SEM. n = 3-4 each group. (**f**) Fresh parametrial fat collected from 15-week-old *Ercc1^-/Δ^* mice was stained for SA-β-gal activity. Representative images taken after 5 hours of staining are shown.

**Figure S3:** (**a**) Quantification of SA-β-Gal^+^ senescent *Zmpste24^-/-^* MPCs and MSCs. Error bars indicate SEM. n = 5 per group. (**b**) Quantification of immunofluorescence staining of γH2AX and fMyHC in *Zmpste24^-/-^* MPCs with or without SR12343 treatment. Error bars indicate SEM. n = 5 per group. (**c**) Quantification of occurrence of nuclear blebbing in *Zmpste24^-/-^* MPCs with or without SR12343 treatment. Error bars indicate SEM. n = 5 per group. (**d**) Quantification of number of SA-β-gal+ senescent cells in skeletal and cardiac muscles of *Zmpste24^-/-^* mice with or without SR12343 treatment. Error bars indicate SEM. n = 5 per group. (**e**) Body weights of *Zmpste24^-/-^* mice with or without SR12343 treatment. (**f**) Quantification of trichrome staining for fibrosis, Pax7+ muscle stem cells, and CD68+ macrophages in skeletal muscles of *Zmpste24^-/-^* mice with or without SR12343 treatment in Fig. 5h. Error bars indicate SEM. n = 5 per group. (**g**) Body weights of WT old mice with or without SR12343 treatment. (**h**) Quantification of Western blotting in Fig. 6d. Error bars indicate SEM. Vehicle, n = 6; SR12343, n = 4. (**i**) Quantification of fiber CSAs from immunohistochemical analysis. Error bars indicate SEM. n = 3 per group.
